# CSEA-Net: A channel–spatial enhanced attention network for lung tumor segmentation on CT images

**DOI:** 10.1016/j.isci.2025.111974

**Published:** 2025-02-25

**Authors:** Wenhu Liu, Jinhao Sun, Han Li, Yan Wang, Zhaohui Wang

**Affiliations:** 1 Department of Cardiology, Union Hospital, Tongji Medical College, Huazhong University of Science and Technology, Wuhan, China; 2 Hubei Key Laboratory of Biological Targeted Therapy, Union Hospital, Tongji Medical College, Huazhong University of Science and Technology, Wuhan, China; 3 Hubei Provincial Engineering Research Center of Immunological Diagnosis and Therapy for Cardiovascular Diseases, Union Hospital, Tongji Medical College, Huazhong University of Science and Technology, Wuhan, China; 4 School of Artificial Intelligence Jilin University, Jilin, China

**Keywords:** Medical imaging, Cancer, Artificial intelligence

## Abstract

The segmentation of lung nodules is a crucial step in the early detection of lung cancer, which remains the leading cause of cancer-related mortality worldwide. To address the need for efficient and accurate diagnosis, we introduce CSEA-Net, a fully automated lung nodule segmentation model designed to operate with high precision on computed tomography images. The model employs a deep learning architecture that incorporates the dual-branch channel–spatial feature enhancement network and a coordinate attention mechanism to address the diagnostic challenges posed by lung nodules that are too small and poorly contoured. CSEA-Net achieves excellent performance in lung tumor segmentation across multiple publicly available datasets with high Dice coefficients and Intersection over Union scores. In conclusion, our proposed model exhibits excellent performance in the accurate segmentation of lung nodules and has the potential to assist doctors in automatically distinguishing the locations of lung tumors.

## Highlights

• An improved deep learning framework for lung nodule segmentation is proposed
• The CSEA module is designed for blurred boundary contours of lung nodules in CT images
• Our model shows outstanding segmentation ability on three well-established public datasets

## Graphical abstract

**Figure:**
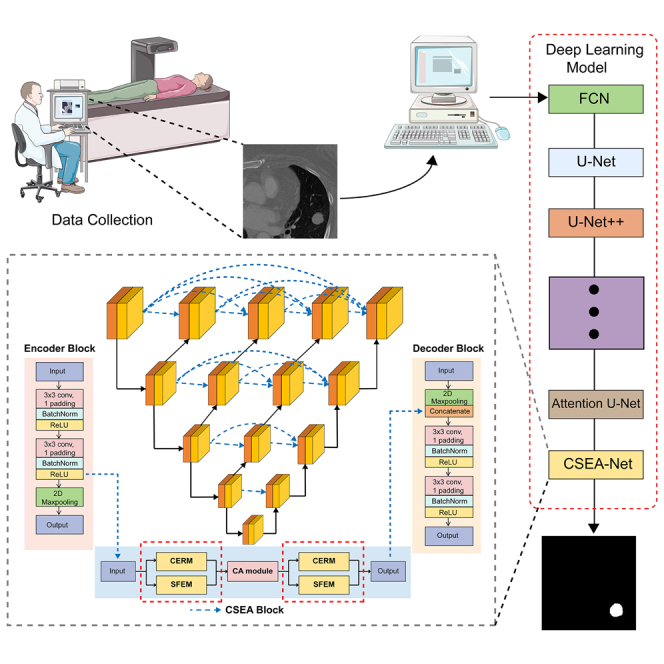

## Introduction

Lung cancer is the leading cause of cancer-related deaths worldwide,^1^ and the prognosis of patients with this malignant tumor is highly dependent on the stage at which it is diagnosed.^2^ However, patients with lung cancer often exhibit only pulmonary nodules in the early stage and have no other obvious symptoms.^3^ Consequently, timely identification of lung nodules plays a crucial role in improving the prognosis of patients.^4^ In clinical practice, lung nodules are predominantly screened through low-dose computed tomography (CT). The segmentation of lung nodules on CT images is a critical process for the early detection and timely treatment of lung cancer.^5^^,^^6^ However, traditional methods of medical image analysis relying on manual segmentation have become progressively unsatisfactory.^7^^,^^8^ One of the challenges in this context is that CT images comprise multiple slices, with nodules exhibiting variable textures, shapes, and locations across different planes.^9^ Nodules may frequently exhibit characteristics that are similar to those of neighboring structures, which can result in unclear boundaries. Furthermore, individual differences among radiologists, such as inexperience in the manual segmentation of lung nodules, may result in susceptibility to misdiagnosis or underdiagnosis. Therefore, replacing traditional lung nodule segmentation with computer-aided diagnostic methods to improve the accuracy of lung nodule detection in patients has become a research priority.

Recently, deep learning, which is successful in that it requires little engineering by hand and new algorithms will accelerate its progress much more,^10^ has demonstrated considerable efficacy in medical image analysis.^11^^,^^12^ Deep learning methods in this field are typically based on U-Net, which facilitates the retention of spatial information and the enhancement of feature extraction through its characteristic U-shaped design and the incorporation of skip connections.^12^ Some modified variants of U-Net, such as Attention U-Net,^13^ U-Net++,^14^ U2-Net,^15^ and R2U-Net,^16^ have been developed to address the shortcomings of U-Net. Despite these advancements, certain limitations remain, including the weak segmentation of small targets and the inability to process detailed information. As a result, current research focuses on creating fully automated and efficient segmentation models to alleviate doctors’ workload and improve the accuracy of lung tumor detection.

To address the aforementioned challenges, we propose CSEA-Net (Figure 4), an automated lung nodule segmentation model that incorporates a channel–spatial enhanced attention (CSEA) module. The architecture of the CSEA module incorporates two dual-branch channel–spatial feature enhancement (DCSE) networks (Figure 5) and a coordinate attention (CA) mechanism (Figure 6) designed to meticulously capture the detailed contours of nodules while increasing the transmission of feature information between the units. The main contributions of this work are as follows.

(1) An improved fully automated model for lung nodule segmentation, CSEA-Net, is proposed. This framework is developed to address the challenge of inaccurate segmentation arising from blurred boundary contours and inconsistent manual segmentation quality.
(2) The DCSE module is designed as a two-branch structure; the upper branch distinguishes features in normal tissue regions and lung nodule regions by using different numbers of convolutional kernels, whereas the lower branch improves the ability of the model to extract features of lung nodule contour information integrity by enhancing the spatial receptive field.
(3) Compared with other models with superior performance in lung nodule segmentation, CSEA-Net demonstrates exceptional performance, as assessed by a comprehensive evaluation on publicly available datasets.

### Related work

#### Lung nodule segmentation

Traditional lung nodule segmentation methods are broadly classified into threshold, region growing, clustering, active contour model, and mathematical model optimization methods.^17^ However, these methods are dependent on manual intervention and are prone to problems such as overly empirical threshold settings, unsatisfactory segmentation of nodules, and excessive computational complexity.^18^

Recently, deep learning methods have achieved excellent performance in the segmentation of lung nodules. The fully connected network (FCN)^19^ was initially proposed in 2015 to address the limitations of traditional convolutional neural networks (CNNs), which are unable to detect and classify images of varying sizes. However, FCNs can segment images of any size. Subsequently, Ronneberger et al.^20^ introduced U-Net, which is based on an encoder–decoder structure. The original skip connection method used in this network combines context information extracted from each encoder layer with abstract semantic information from the decoder layer. This approach significantly increases segmentation accuracy and has since become a cornerstone technique in medical image segmentation.

Subsequent studies have employed U-Net as a foundation for further enhancements in diverse segmentation tasks. Zhou et al.^14^ extended the U-Net architecture by introducing an information flow mechanism that connects different layers; this model is called U-Net++, and it can be used for more accurate multiscale segmentation of lung lesions appearing in images. GUNet3++^21^ is a variant of the U-Net architecture that uses an evolutionary algorithm and outperforms the baseline in lung nodule segmentation. Wang et al.^22^ reported a multi-view CNN for lung nodule segmentation that is designed to simultaneously extract different nodule features from axial, coronal and sagittal CT image views. The scale-aware multi-attention guided reversal network (SM-RNet)^23^ employs a weighted cross-scale fusion technique for features with different scale attentions that focuses on specific regions and details, resulting in superior performance on the LUNA16 dataset. However, although these CNN-based segmentation methods have achieved better accuracy through various improvements, the ability to learn the semantic features of planar images is still limited. Furthermore, these methods lack both the capacity to extract data features and sensitivity to feature changes caused by graphical deformation.

#### Attention mechanism

The attention mechanism is a computational technique designed to imitate the attention of humans. This mechanism is capable of rapidly filtering out high-value information from a vast quantity of data and has been extensively employed in numerous deep learning models within the domain of image processing.^24^^,^^25^ The SENet model^26^ incorporates squeeze-and-excitation (SE) blocks to increase the focus on essential feature channels by dynamically adjusting the weights of each channel, consequently increasing the model’s performance. Oktay et al.^13^ integrated the attention gate module into the U-Net architecture to create the Attention U-Net network, which enables automatic learning of target structures of various shapes and sizes. This integration minimizes computational complexity while enhancing sensitivity and computational accuracy. Spatial Attention-UNet (SA-UNet)^27^ incorporates a spatial attention module combined with dropout convolutional blocks to dynamically enhance features and prevent overfitting. The convolutional block attention module (CBAM)^28^ is a versatile and lightweight attention mechanism that seamlessly integrates into any CNN architecture. The CBAM calculates the attention map in sequence, considering both channel and spatial dimensions, and then adjusts the input feature map by multiplying it with the attention map for optimization. Hou et al.^29^ improved the U-Net structure by integrating self-attention combined with residual structure and multi-scale features to form the MSR-UNet model, which achieved remarkable performance in lung nodule segmentation.

#### Feature enhancement

The extraction and enhancement of effective features from raw images is a significant challenge in the field of computer vision, and improvements in this process have the potential to enhance model performance.^30^ The feature pyramid network (FPN),^31^ which uses a top-down architecture with lateral connections, is an inaugural attempt to enhance the representational capabilities of CNNs by fusing different levels of features and constructing a feature pyramid. In contrast to the FPN, the attention aggregation feature pyramid network (A2-FPN)^32^ uses attention-guided feature aggregation to improve multiscale feature learning, aiming to overcome intrinsic deficiencies in feature extraction and fusion. Luo et al.^33^ introduced a channel-enhanced feature pyramid network (CE-FPN) incorporating a subpixel skip fusion module, a subpixel context enhancement module, and a channel attention guided module, with the aim of mitigating the inherent limitations of channel reduction. By extending the information flow path, the multi-branch model enables accurate image segmentation by fusing multiple branch networks.^34^^,^^35^ Trident Net^36^ was proposed design to utilize the receptive field effect for object detection across different scale sizes. Using a parallel multi-branch architecture, this design guarantees that no additional parameters or computations are needed during the forward inference process. Jha and colleagues^37^ introduced an improved architecture called DoubleU-Net, which combines two U-Net architectures. The objective of this design is to increase the efficiency of medical image segmentation by capturing semantic information.

These relevant works are summarized in Table 1.

**Table 1:** Summary of the relevant works

Types	Models	Years	Advantages	Limitations	References
Lung nodule segmentation	FCN	2015	End-to-end, able to handle image inputs of any size, fast processing speeds	No consideration of pixel-to-pixel relationships, segmentation results not sufficiently granular	Longet al.^19^
	U-Net	2015	Able to handle image inputs of any size, low dependence on labeled data, strong feature extraction capability	High computational requirements, poor segmentation of small targets, poor processing of detailed information	Ronneberger et al.^20^
	U-Net++	2020	Flexible network structure with deep supervision, improved accuracy through integration of different feature levels	Introduces more parameters and takes up more internal reference space	Zhou et al.^14^
	GUNet3++	2023	Introduction of evolutionary algorithms, higher segmentation accuracy than U-Net++	Requires more effort for both training and inference	Ardimento et al.^21^
	MV-CNN	2017	Introduction of multi-scale information	Inadequate segmentation accuracy	Wanget al.^22^
	SM-RNet	2023	Combination of global contextual and spatial information from small-scale and large-scale features, higher segmentation accuracy than U-Net++	Performance still has room for improvement	Tang et al.^23^
Attention mechanism	SENet	2018	Introduction of channel attention, Squeeze and Excitation operations, Plug-and-Play	More complex network structure, higher requirements for computing resources and memory	Hu et al.^26^
	Attention U-Net	2018	Introduction of attention gates to the skip connections to U-Net, Plug-and-Play	Increase in training parameters and computational cost	Oktay et al.^13^
	SA-UNet	2021	Introduction of spatial attention, lightweight network	No consideration of channel dimension information	Guo et al.^27^
	CBAM	2018	Combination of spatial attention and channel attention, Plug-and-Play	Requires more computational resources, higher computational complexity	Woo et al.^28^
	SMR-UNet	2023	Integration of self-attention, multi-scale features and residual structures	Performance still has room for improvement	Hou et al.^29^
Feature enhancement	FPN	2017	Multi-scale feature fusion, computationally efficient, flexible embedding	Relatively complex network structure, certain requirements on hardware resources	Lin et al.^31^
	A2-FPN	2022	Improved multi-scale feature learning through attention-guided feature aggregation	Increased computational complexity	Hu et al.^32^
	CE-FPN	2021	Alleviation of channel information loss and the aliasing effects, lightweight network, improved performance of the FPN-based networks	Some inference time increases	Luo et al.^33^
	Trident Net	2019	Ability to generate scale-specific feature maps with a uniform representational power through multi-branch structure and scale-aware training	Slow inference speed due to its branching nature	Li et al.^36^
	DoubleU-Net	2020	Two U-Net architectures in sequence with two encoders and two decoders, better performance than U-Net	Uses more parameters and requires increased training time	Jha et al.^37^

## Results

### Datasets and data preprocessing

#### LUNA16 dataset

The LUNA16 dataset^38^ is derived from the Lung Image Database Consortium and Image Database Resource Initiative (LIDC-IDRI), the most extensive publicly accessible database of lung CT images. The LUNA16 dataset comprises CT image data of 888 cases with annotations, which contain information on the coordinates of the nodes.

#### MSD dataset

The Medical Segmentation Decathlon (MSD) lung dataset^39^ consists of thin-section CT scans obtained from 96 patients diagnosed with non-small cell lung cancer acquired with the following parameters: slice thickness, less than 1.5 mm; automatic tube current modulation, 100–700 mA; tube rotation speed, 0.5 s; helical pitch, 0.9–1.0; and reconstruction via a sharp kernel.

#### LNDb dataset

The LNDb dataset^40^ comprises 294 lung CT scans obtained from the Centro Hospitalar e Universitário de São João (CHUSJ) in Porto, Portugal, from 2016 to 2018. Each scan is meticulously annotated according to the LIDC-IDRI methods to facilitate the identification and characterization of lung nodules and suspected lesions.

To ensure consistency, the same data preprocessing steps are performed on these datasets. The CT values of each image are clipped to the range of [−1000, 800] Hu. The datasets are subsequently preprocessed via random selection techniques, including HSV color space conversion and luminance contrast adjustment, followed by normalization for model training purposes. To accentuate the lung region, we extract four patches of size 224 × 224 from the lung images through a restrictive cropping method that focuses on specific regions of interest. The images are divided into a training set, a test set and a validation set at a ratio of 7:2:1. This division enables a thorough assessment of the segmentation methods across diverse conditions. The datasets utilized in the experiments are outlined in Table 2.

**Table 2:** Summary of the medical image segmentation datasets utilized in our experiments

Dataset	Modality	Segmentation	Cases	Input size	Images	Division ratio
LUNA16	CT	Lung nodule	808	3∗224∗224	9757	7:2:1
MSD	CT	Lung nodule	96	3∗224∗224	1228	7:2:1
LNDb	CT	Lung nodule	294	3∗224∗224	1226	7:2:1

Images: the number of preprocessed input images; division ratio: the ratio of training, testing, and validation sets.

### Loss function

Since the small areas of the nodules lead to chaotic training error curves using the Dice loss function, we use this function in conjunction with the binary cross-entropy (BCE) loss function, which can stably segment small target objects. The proposed loss function formula is shown in Equations 1, 2, and 3, where *x* denotes the gradient coefficient of BCE loss and λ denotes the Dice coefficient of the foreground object that is the focus of the Dice loss function.

(Equation 1) $$\begin{array}{c} L_{\text{Dice}}=1-\frac{2\left|X\cap Y\right|+\lambda }{\left|X\right|+\left|Y\right|+\lambda }\; \end{array}$$

(Equation 2) $$L_{BCE}=\text{ylog}\left(x\right)+\left(1-y\right)\log\left(1-x\right)$$

(Equation 3) $$\begin{array}{c} L=0.5L_{Dice}+L_{BCE} \end{array}$$

### Evaluation metrics

For quantitative analysis of the experimental results, the Intersection over Union (IoU) and Dice similarity coefficient (DSC) values are determined. Equations 4 and 5 are used to calculate the overall IoU and DSC, with *TP* representing the number of true positives, *FP* representing false positives, and *FN* representing false negatives.

(Equation 4) $$\begin{array}{c} IOU=\frac{TP}{TP+FN+FP} \end{array}$$

(Equation 5) $$\begin{array}{c} DSC=\frac{2\cdot TP}{2\cdot TP+FN+FP} \end{array}$$

In addition, we introduce boundary IoU,^41^ a new segmentation evaluation measure focused on boundary quality, represented in Equation 6:

(Equation 6) $$\begin{array}{c} Boundary\;IoU\left(G,P\right)=\frac{\left|\left(G_{d}\cap G\right)\cap \left(P_{d}\cap P\right)\right|}{\left|\left(G_{d}\cap G\right)\cup \left(P_{d}\cap P\right)\right|} \end{array}$$

where G and P represent the ground truth binary mask and prediction binary mask, respectively; d represents the pixel width of the boundary region; and $G_{d}$ and $P_{d}$ represent the set of pixels in the boundary regions of the binary masks.

The boundary IoU quantifies the overlap between the predicted boundary and the ground truth boundary, thereby aiding in a more accurate evaluation of the model performance in extracting lung nodule boundaries.

### Performance quantification

CSEA-Net is compared with the preeminent approaches in the medical imaging domain, including FCN, U-Net, U2-Net, R2U-Net, U-Net++, SAM-UNet,^42^ CAM-UNet,^43^ CBAM-Unet++,^44^ and Attention U-Net. The evaluation metrics employed are IoU, DSC, BCE-Dice loss, and boundary IoU. Table 3 summarizes the results of the comparisons.

**Table 3:** Quantitative results of our CSEA-Net and other models on various datasets

Dataset	Model	IoU (%)	DSC (%)	BCE-Dice loss	Boundary IoU
LUNA16	FCN	81.34	89.47	0.1276	74.36
	U-Net	82.81	90.46	0.1041	75.87
	U2-Net	82.24	90.11	0.1111	73.49
	R2U-Net	82.02	89.73	0.1141	74.29
	U-Net++	82.54	90.19	0.1063	76.63
	SAM-UNet	82.38	89.92	0.1085	77.92
	CAM-UNet	82.93	90.32	0.1072	76.05
	CBAM-Unet++	83.13	91.12	0.0979	78.17
	Att-U-Net	83.4	90.73	0.1064	77.41
	CSEA-Net (ours)	86.56	92.69	0.079	82.16
MSD	FCN	89.25	93.96	0.0734	82.52
	U-Net	85.7	91.92	0.0951	81.54
	U2-Net	81.81	89.53	0.1247	77.06
	R2U-Net	88.67	93.58	0.0789	82.37
	U-Net++	91.34	95.15	0.0558	87.64
	SAM-UNet	89.76	93.79	0.0742	87.39
	CAM-UNet	90.31	94.12	0.0638	85.49
	CBAM-Unet++	91.87	95.37	0.0529	87.92
	Att-U-Net	83.83	90.82	0.1092	80.51
	CSEA-Net (ours)	95.03	97.17	0.0363	89.26
LNDb	FCN	93.19	96.31	0.0449	87.99
	U-Net	92.24	95.78	0.0493	88.12
	U2-Net	89.59	94.29	0.0783	82.70
	R2U-Net	89.42	94.21	0.0792	84.33
	U-Net++	94.33	96.94	0.0365	89.84
	SAM-UNet	92.89	95.73	0.0496	88.17
	CAM-UNet	93.76	96.06	0.0452	87.93
	CBAM-Unet++	95.19	97.17	0.0341	89.59
	Att-U-Net	91.44	95.35	0.0567	85.58
	CSEA-Net (ours)	97.58	98.65	0.015	92.03

Att-U-Net: Attention U-Net.

On all three datasets, CSEA-Net outperforms the other models in all metrics, with DSCs of 92.69% for LUNA, 97.17% for MSD, and 98.65% for LNDb. In addition, CSEA-Net achieves an IoU of 86.56% in LUNA, 95.03% in MSD, and 97.58% in LNDb. The BCE-Dice loss in these three datasets is also significantly superior to those of the other models (0.079 in LUNA, 0.0363 in MSD, and 0.015 in LNDb). In addition, the boundary IoU of CSEA-Net outperforms those of the other models, which demonstrates the superior performance of our model in dealing with the blurred boundary contours of lung nodules. These results indicate that CSEA-Net results in a greater degree of overlap between predictive segmentation and real-world segmentation and has a more pronounced advantage and better performance in handling medical image details.

Compared with other models, U-Net++ achieves exceptional performance, with DSCs of 90.19% in LUNA, 95.15% in MSD, and 96.94% in LNDb. However, the performance of our model compared with U-Net++ improves by 2.5%, 4.02%, and 2.73% for DSC, IoU and BCE-Dice loss, respectively, on the LUNA16 dataset; 2.02%, 3.67% and 1.95% on the MSD dataset; and 2.34%, 3.25%, and 2.15% on the LNDb dataset. These improvements may be attributed to the attention mechanism and advanced feature extraction strategies inherent to these networks.

The combined findings from this experiment suggest that the CSEA-Net model, which incorporates a feature enrichment framework and a CA mechanism, is clearly superior to other models in segmenting lung tumor images.

### Qualitative performance

**Figure 1:**
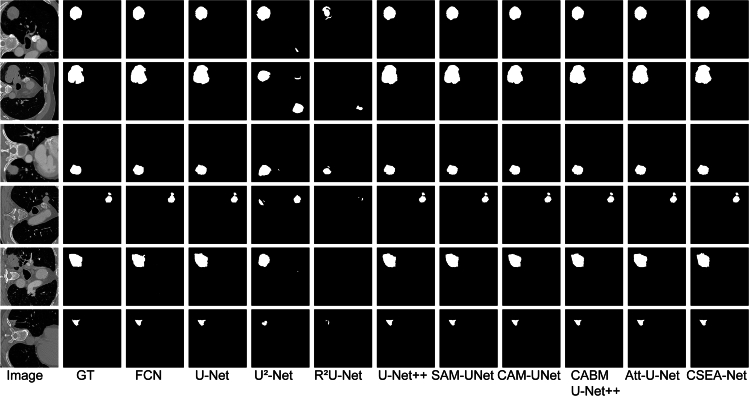
Comparison of qualitative results from various methods on the LUNA16 dataset GT: ground truth; Att-U-Net: Attention U-Net.

**Figure 2:**
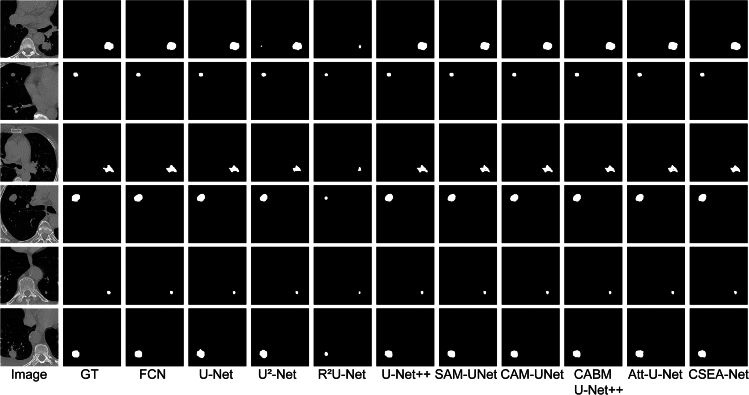
Comparison of the qualitative results from various methods on the MSD dataset GT: ground truth; Att-U-Net: Attention U-Net.

**Figure 3:**
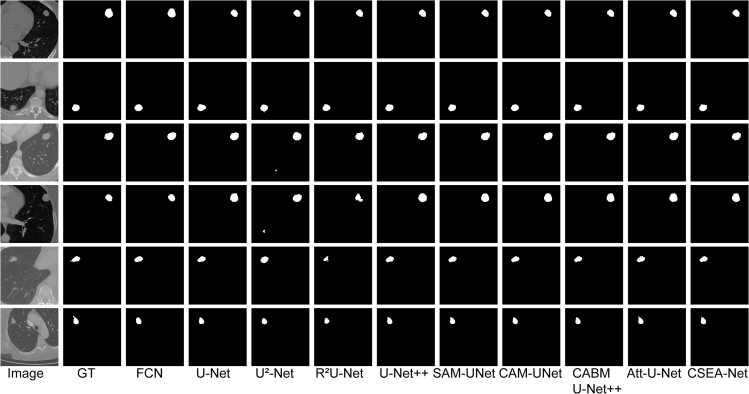
Comparison of the qualitative results from various methods on the LNDb dataset GT: ground truth; Att-U-Net: Attention U-Net.

The results of the qualitative assessment of the segmentation are presented in Figures 1, 2, and 3. The FCN, U-Net, U2-Net and R2U-Net architectures generally enable the segmentation of lung nodules but have limitations in accurately depicting tumor boundaries. Attention U-Net and U-Net++ have been improved by the addition of attention mechanisms and volume processing and achieve results closer to real data, but these models still suffer from output fragmentation and contour inaccuracies according to our results.

The excellent performance of CSEA-Net is attributed to the addition of spatial and channel feature enhancement modules with the CA mechanism. These components enable the model to capture the complex morphology of the tumor and reduce feature incompatibilities, allowing CSEA-Net to approximate the ground truth with excellent accuracy on all three datasets.

The qualitative analysis indicates that CSEA-Net offers enhanced capabilities for processing medical image details on all three datasets, substantiating its robustness in lung tumor segmentation.

### Ablation study

Ablation experiments are performed by removing different modules of the proposed model to validate the segmentation capability. The ablation experiments depict the incremental integration of the DCSE module and CA module into the U-Net++ structure, elucidating their individual and collective contributions to segmentation accuracy. The details are presented in Table 4.

**Table 4:** Ablation studies on each dataset

Dataset	Model	IoU (%)	DSC (%)	BCE-Dice loss
LUNA16	U-Net++	82.54	90.19	0.1063
	U-Net++ with DCSE	82.24	90.11	0.1111
	U-Net++ with CA	83.17	91.24	0.0964
	CSEA-Net (ours)	86.56	92.69	0.079
MSD	U-Net++	91.34	95.15	0.0558
	U-Net++ with DCSE	90.43	94.28	0.0742
	U-Net++ with CA	93.26	96.24	0.0473
	CSEA-Net (ours)	95.03	97.17	0.0363
LNDb	U-Net++	94.33	96.94	0.0365
	U-Net++ with DCSE	94.26	96.75	0.0384
	U-Net++ with CA	95.47	97.12	0.0324
	CSEA-Net (ours)	97.58	98.65	0.015

DCSE: dual-branch channel–spatial feature enhancement network, CA: coordinate attention module.

Following the introduction of the dual-channel module to the U-Net++ framework, the DSC has not significantly improved. This result occurs because the DCSE network introduces a considerable number of parameters to U-Net++, resulting in the network becoming consistently heavy. On the other hand, integrating the CA module to increase the model’s attention significantly increases both the DSC and IoU, underscoring the critical role of capturing viewpoint information in enhancing the segmentation accuracy.

The ablation study highlights the synergistic effect of integrating the dual-branch enhanced feature convolutional module and CA mechanism into the U-Net++ framework on enhancing lung tumor segmentation. The incorporation of these modules into the U-Net++ architecture results in a significant improvement in performance and more accurate delineation of the tumor boundaries.

## Discussion

The application of deep learning in the accurate segmentation of lung nodules has gradually superseded traditional methods, representing a significant advancement in the diagnosis and treatment of lung cancer.^45^ However, deep learning via neural network models still faces challenges, including network convergence issues, a lack of generalizability, low segmentation accuracy and efficiency, an inability to achieve fully automated segmentation, a limited ability to extract data features, and low sensitivity to feature changes due to graphical deformation.^46^ On this basis, we design CSEA-Net and conduct a comprehensive evaluation of its performance in lung cancer image segmentation on publicly available lung tumor datasets.

In this study, we comparatively analyze seven different segmentation methods. The results show that CSEA-Net is an effective segmentation network for lung nodules, outperforming existing models while maintaining the integrity of the edges of tiny nodules. In addition, CSEA-Net is a completely automated system that can perform image segmentation without human involvement. The proposed CSEA-Net integrates spatial and channel feature enhancement modules with an attention mechanism. These additions allow the model to capture intricate details and enhance its focus on the image, thereby extracting more valuable features. The efficacy of this architecture is demonstrated through validation on a range of datasets, all of which demonstrate superior performance to current approaches.

### Limitations of the study

Despite the promising results demonstrated by CSEA-Net in lung tumor segmentation, there are still several limitations that require further attention. The results presented here are contingent upon the availability of high-quality annotated datasets, and the performance of the model on extremely heterogeneous tumors or tumors with very low contrast with the surrounding tissue has not been extensively tested. Furthermore, the present study is conducted exclusively on 2D medical images, and the applicability and performance of CSEA-Net on 3D medical images still need to be explored. Future work will aim to improve the robustness of the model to accommodate more diverse datasets of different sizes, as well as to optimize the model without sacrificing accuracy. Some important techniques, such as auto-image augmentations,^47^ will be explored to improve the segmentation performance of the model. Additionally, the model will be extended to 3D lung nodule segmentation for clinical applications.

### Conclusion

In conclusion, we propose an improved segmentation network, CSEA-Net, for the automatic segmentation of lung nodules with ambiguous boundaries, variable sizes and unpredictable growth locations on lung CT images. Our proposed method achieves excellent performance in lung nodule segmentation, outperforming other superior-performing models in a comprehensive evaluation on multiple publicly available datasets. Therefore, our proposed method enables the automated identification of lung tumors in clinical applications and can reduce the burden of annotating CT scans by radiologists.

## Resource availability

### Lead contact

Further information and requests for resources and reagent information should be directed to and will be fulfilled by the lead contact Zhaohui Wang (wuxiaohongtian@163.com).

### Material availability

This study did not generate new unique reagents.

### Data and code availability

1. The source code employed in the current research can be accessed on the GitHub page: https://github.com/liuwenhu163/lung_cancer.
2. The datasets analyzed in this paper (LUNA16, MSD, LNDb) are taken from existing publicly available datasets. The DOIs are listed in the key resources table.
3. Any additional information required to reanalyze the data reported in this paper is available from the lead contact upon request.

## Acknowledgments

This research received financial support from the 10.13039/501100001809National Natural Science Foundation of China (No. 82070400 and No.82270367).

## Author contributions

**W.L.:** Writing–original draft, data curation, and conceptualization. **J.S.:** Software, methodology, and conceptualization. **H.L.:** Writing–review and editing and conceptualization. **Y.W.:** Writing–review and editing and project administration. **Z.W.:** Writing–review and editing, project administration, supervision, and funding acquisition.

## Declaration of interests

The authors declare no competing interests.

## STAR★Methods

### Key resources table

**Table:** 

REAGENT or RESOURCE	SOURCE	IDENTIFIER
**Deposited data**		

LUNA16	Setio et al.^38^	https://luna16.grand-challenge.org/
MSD	Antonelli et al.^39^	http://medicaldecathlon.com/
LNDb	Pedrosa et al.^40^	https://lndb.grand-challenge.org/

**Software and algorithms**		

Python version 3.7	Python Software Foundation	https://www.python.org/
PyTorch version 1.8	Facebook	https://pytorch.org/
PyCharm version 2022.3.3	Python IDE	https://www.jetbrains.com/pycharm/
FCN	Long et al.^19^	https://ieeexplore.ieee.org/document/7298965
U-Net	Ronneberger et al.^20^	https://doi.org/10.1007/978-3-319-24574-4_28
U2-Net	Qin et al.^15^	https://doi.org/10.1016/j.patcog.2020.107404
R2U-Net	Zahangir et al.^16^	https://doi.org/10.48550/arXiv.1802.06955
U-Net++	Zhou et al.^14^	https://ieeexplore.ieee.org/document/8932614
SAM-UNet	Yang et al.^42^	https://doi.org/10.48550/arXiv.2408.09886
CAM-UNet	Lin et al.^43^	https://doi.org/10.1016/j.knosys.2021.107272
CBAM-Unet++	Zhao et al.^44^	https://ieeexplore.ieee.org/document/9622008
Att-U-Net	Oktay et al.^13^	https://doi.org/10.48550/arXiv.1804.03999

### Experimental model and study participant details

This study is computational science research and does not utilize experimental models typical of the life sciences.

### Method details

#### Network architecture

**Figure 4:**
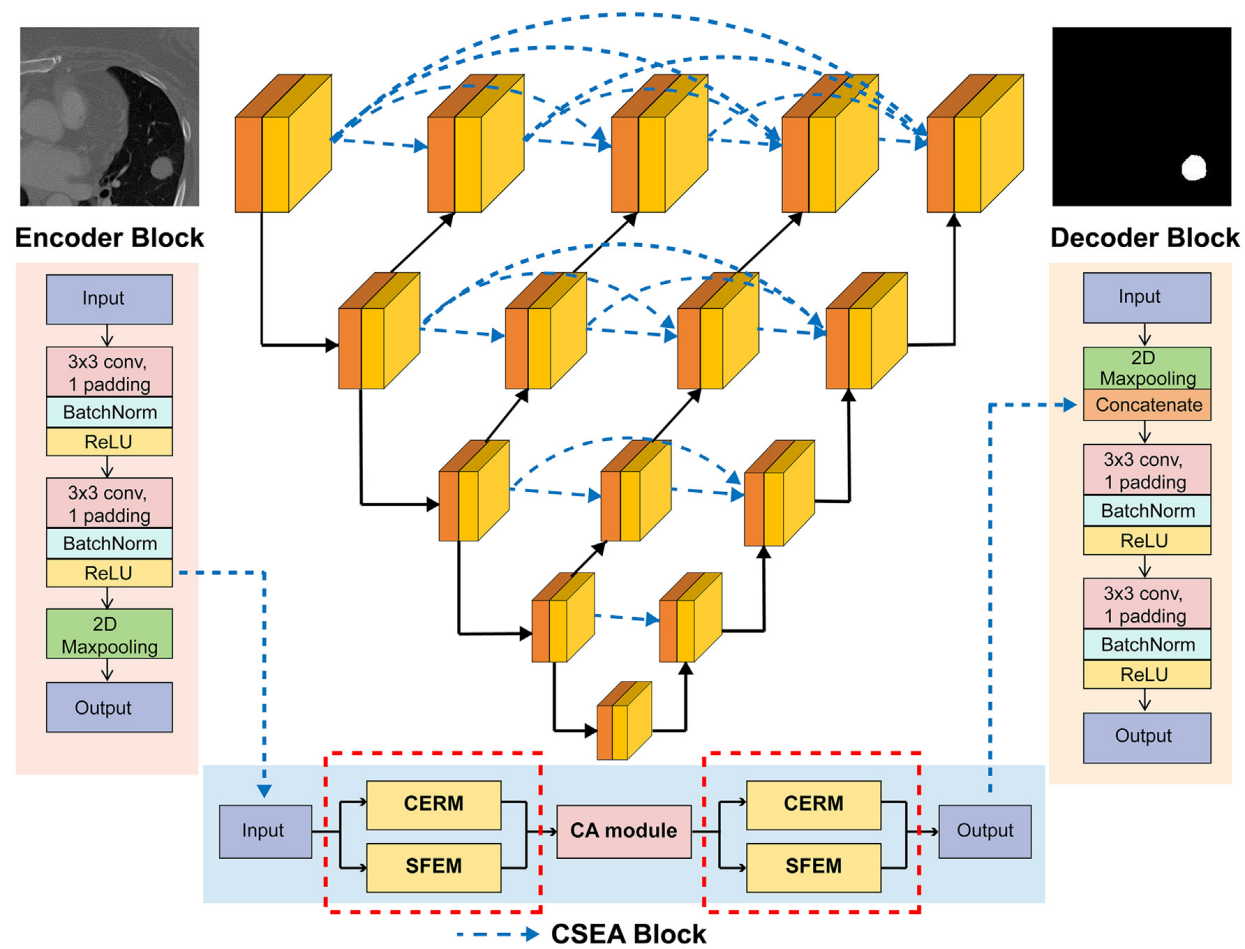
Comprehensive architecture of CSEA-Net for lung tumor segmentation CSEA-Net employs the encoding and decoding layers of U-Net++ with improvements in skip connections. The CSEA block replaces the skip connection with three components: (1) the channel-enhanced residual module (CERM); (2) the spatial feature enhancement module (SFEM); and (3) the coordinate attention (CA) module. The combination of the CERM and SFEM forms a dual-branch channel–spatial feature enhancement network, which enables more comprehensive capture of features. The CA module is designed to filter the extensive features to identify those that are valid.

The CSEA-Net architecture enhances segmentation performance in medical image analysis by incorporating the CSEA module to optimize the skip connection structure in UNET++, enabling more efficient feature extraction and broadcasting. A visual representation of our framework is shown in Figure 4.

CSEA-Net employs an encoder and a decoder, which are interconnected via skip connections, to delineate the regions of lung nodules within a CT scan image of size 224×224. The encoder block extracts feature from the image and consists of two convolutional layers with a 3×3 kernel size, batch normalization, and ReLU activation, as well as a pooling layer that achieves downsampling with a 2×2 stride. The decoder is meticulously crafted to restore the spatial resolution of the image and includes an upsampling layer that receives and concatenates the corresponding feature maps from the encoder, followed by two convolutional layers with batch normalization and ReLU activation for subsequent refinement. The CSEA block, which replaces the skip connection, transfers the feature map from the secondary ReLU activation of the encoder block to the upsampling layer of the decoder block.

The CSEA block addresses the shortcomings of the skip connection in U-Net++ by incorporating three components as follows: (1) a channel-enhanced residual module (CERM); (2) a spatial feature enhancement module (SFEM); and (3) a coordinate attention (CA) module. The CSEA block first performs feature enhancement through the first DCSE module, in which the upper branch (CERM) uses different numbers of convolution kernels and stacked residual convolution modules to increase the diversity of channel features, and the lower branch (SFEM) uses dilated convolution to increase the model's receptive field. The subsequent CA mechanism filters the features extracted by the DCSE module to increase the model's sensitivity to the target location and shape. Finally, the filtered features are further enhanced by the second DCSE module. Through these operations, the CSEA module finely learns the location and shape of the nodule, which results in a clear segmentation of the lung nodule contour.

#### Channel-enhanced residual module

**Figure 5:**
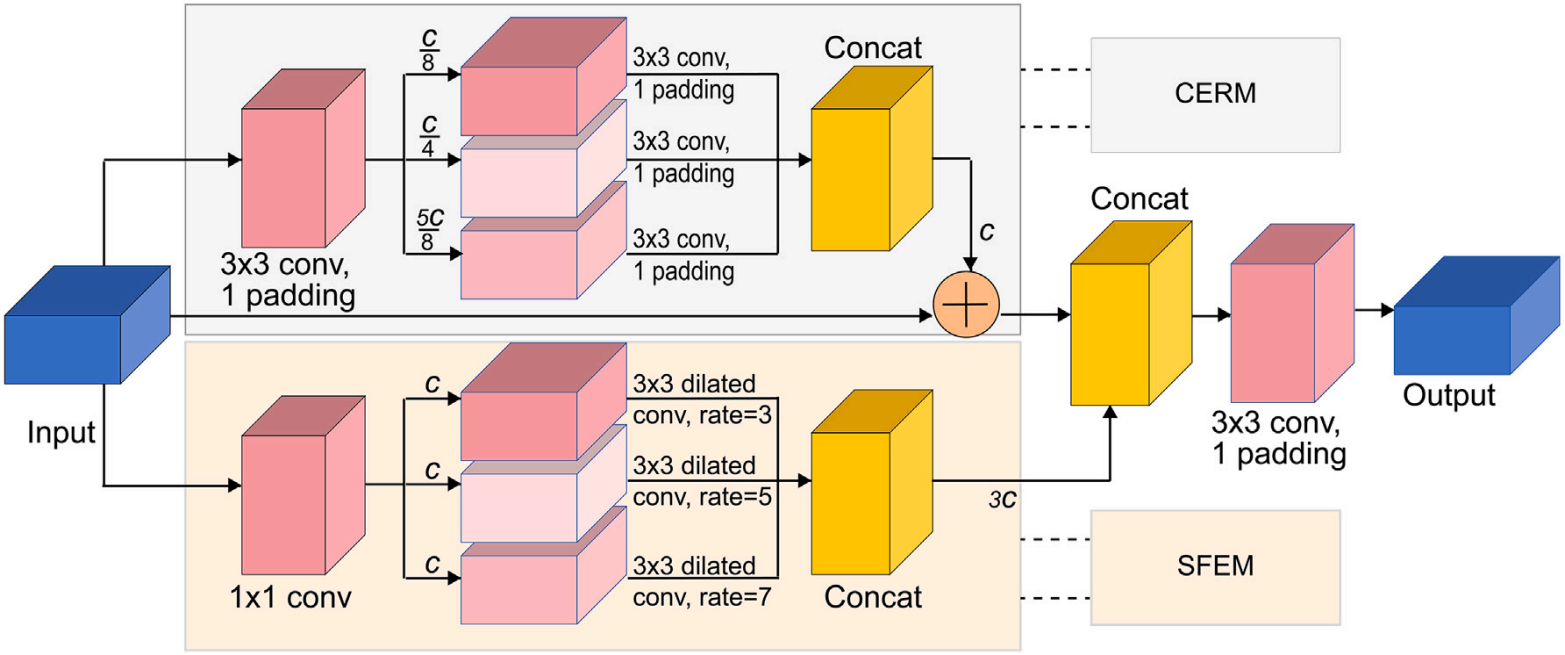
Architectural design of the dual-branch channel–spatial feature enhancement (DCSE) network The DCSE network consists mainly of the CERM for extracting channel information and the SFEM for extracting spatial information. CERM: channel enhanced residual module, SFEM: spatial feature enhancement module.

The quality of channel feature extraction is closely related to the segmentation effect of the model, so we add the CERM to our model, as shown in Figure 5. The CERM initially conducts a 3×3 convolution operation with the number of filters C on the accepted input X.

(Equation 7) $$\begin{array}{c} x_{1}=F\left(x,C\right) \end{array}$$

To enlarge the channel information, we use a 3×3 convolution with three different filters of multiple channel scales with filter numbers of C/8, C/4, and 5C/8. The features obtained with different filter numbers are then fused via a concatenation operation, followed by residual concatenation to obtain additional spatial features of the nodes. The residual operation also allows our channel-enhanced residual block to capture the network more accurately and learn the object of interest. The output can be expressed with the following equation:

(Equation 8) $$\begin{array}{c} y_{1}=x+Concat\left(F\left(x_{1},\alpha C\right),F\left(x_{1},2\alpha C\right),F\left(x_{1},5\alpha C\right)\right)\text{,} \end{array}$$

where α =1/8 is a constant and *F* denotes a convolutional transform function.

The CERM optimizes the efficiency of lung channel information extraction by incorporating various kernel sizes and residual structures, resulting in improved model performance.

#### Spatial feature enhancement module

Increasing the receptive field can improve the learning effect of the model on the edge contour of the object. The SFEM uses dilated convolution^48^ to improve the receptive field of the model, as illustrated in Figure 5. Following the initial processing of the input features through a 1×1 convolution, additional spatial information is obtained by applying 3×3 dilated convolutions with varying dilation rates (r1 = 3, r2 = 5, r3 = 7). These dilated features are subsequently fused to obtain spatial enhancement features. These transformations are represented by the following equations:

(Equation 9) $$\begin{array}{c} x_{1}=F\left(x,C\right)\text{,} \end{array}$$

(Equation 10) $$\begin{array}{c} y_{2}=Concat\left(F_{dilated}\left(x_{1},r_{1}\right),F_{dilated}\left(x_{1},r_{2}\right),F_{dilated}\left(x_{1},r_{3}\right)\right)\text{,} \end{array}$$

where *F* denotes a convolutional transform function and *F*_*dilated*_ represents a dilated convolutional transform function.

The SFEM enhances the convolution process to capture the feature map and extract features of larger size through different receptive fields, thus achieving spatial information enhancement.

The DCSE network fuses the channel-enhanced features obtained by the CERM with the spatially enhanced features obtained by the SFEM to obtain output features that contain rich contextual and localization information of the lung nodules. The output features can be written as follows:

(Equation 11) $$\begin{array}{c} y=\text{Concat}\left(y_{1},y_{2}\right) \end{array}$$

#### Coordinate attention module

Channel attention has a significant effect on improving model segmentation performance, but it usually ignores location information, which is important for generating spatially selective attention maps. For this reason, we adopt CA,^49^ which is effective in capturing long-range dependencies and preserving cross-channel information.

**Figure 6:**
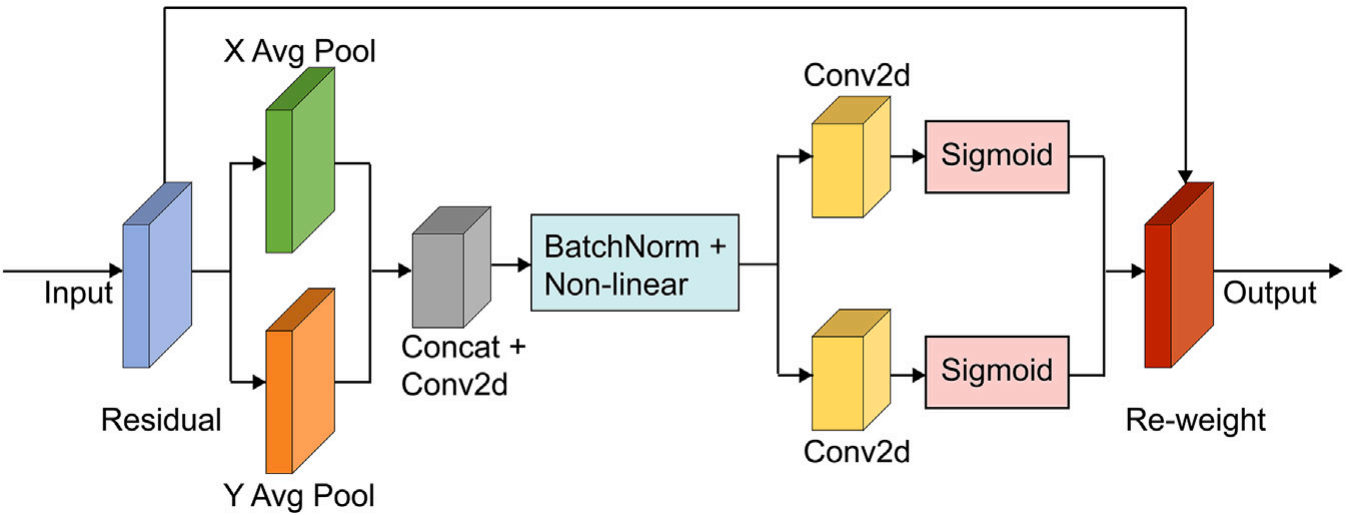
Architecture of the coordinate attention module

As depicted in Figure 6, given the input Y, the CA module encodes each channel along both horizontal and vertical coordinates by employing a pooling kernel with dimensions (H, 1) or (1, W). The output of the c-th channel at height h and width w can be computed with Equations 12 and 13.

(Equation 12) $$\begin{array}{c} z_{c}^{h}\left(h\right)=\frac{1}{W}\sum_{0\leq i<W}y\left(h,i\right) \end{array}$$

(Equation 13) $$\begin{array}{c} z_{c}^{w}\left(w\right)=\frac{1}{H}\sum_{0\leq j<H}y\left(j,w\right) \end{array}$$

The two 1D direction-aware features, *z*^*h*^ and z^*w*^, are concatenated, followed by a 1×1 convolution, and then batch normalization and non-linear transformation are applied, as specified in Equation 14.

(Equation 14) $$\begin{array}{c} f=\delta \left(F_{1}\left(Concat\left(z^{h},z^{w}\right)\right)\right)\text{,} \end{array}$$

where *δ* represents a non-linear activation function, *F*_1_ denotes a convolutional transform function, and *f* is an intermediate feature mapping that encodes spatial information in the horizontal and vertical directions.

Then, *f* is decomposed along the spatial dimensions into 2 separate tensors *f* ^*h*^ ∈ R^C/r x H^ and *f* ^w^∈ R^C/r x W^, which are subsequently transformed into tensors with the same number of channels using two additional 1×1 convolutional transforms *F* ^h^ and *F* ^w^. The sigmoid activation function *σ* is then used to calculate the weights in the horizontal and vertical directions, which are denoted as follows:

(Equation 15) $$\begin{array}{c} g^{h}=\sigma \left(F_{h}\left(f^{h}\right)\right)\text{,} \end{array}$$

(Equation 16) $$\begin{array}{c} g^{w}=\sigma \left(F_{w}\left(f^{w}\right)\right)\;\text{.} \end{array}$$

Finally, the output *U* can be written as follows:

(Equation 17) $$\begin{array}{c} u_{c}\left(i,j\right)=y_{c}\left(i,j\right)\cdot g_{c}^{h}\left(i\right)\cdot g_{c}^{w}\left(j\right)\;\text{.} \end{array}$$

We adapt the CA module to reweight the spatial feature of the dual-branch channel–spatial module, and the reweighted enhanced feature has the ability to learn more contour shapes of the lung nodule.

#### Implementation details

The Adam optimization function, which incorporates a weight decay of 1e-6 and uses small batch sizes of 4 samples, is applied to compute weight gradient updates. The initialization of the network's weights follows the previously proposed method,^50^ and the training proceeds for 300 epochs, with an initial learning rate of 1e-3 that is subsequently adjusted by cosine annealing.^51^ The PyTorch open-source framework is used to conduct experiments. All training and validation procedures for the models are executed on an NVIDIA 4090 GPU with 24 GB of memory within a 16-core, 48-thread server featuring an Intel(R) Xeon(R) Gold 6430 (128 GB RAM).

### Quantification and statistical analysis

The metrics analysis for calculating IoU, DSC, BCE-Dice loss, and Boundary IoU was conducted using Python (version 3.7, https://www.python.org/).

### Additional resources

This study did not create or expand any websites or resources, and it does not involve clinical experiments.
